# Diabetes mellitus im Kindes- und Jugendalter (Update 2026)

**DOI:** 10.1007/s00508-025-02650-3

**Published:** 2026-04-30

**Authors:** Birgit Rami-Merhar, Elke Fröhlich-Reiterer, Sabine E. Hofer, Dagmar Meraner, Katrin Nagl, Maria Fritsch

**Affiliations:** 1 Universitätsklinik für Kinder- und Jugendheilkunde, Abteilung für Pulmonologie, Allergologie und Endokrinologie, Medizinische Universität Wien, Wien, Österreich; 2 Universitätsklinik für Kinder- und Jugendheilkunde, Abteilung für Allgemeine Pädiatrie, Medizinische Universität Graz, Graz, Österreich; 3 Universitätsklinik für Pädiatrie 1, Medizinische Universität Innsbruck, Innsbruck, Österreich

**Keywords:** Typ 1 Diabetes, Metabolische Kontrolle, Schwere Hypoglykämien, AID – automatische Insulindosierung, Multidisziplinäre Betreuung, Type 1 Diabetes, Metabolic control, Severe hypoglycemia, AID—automated insulin dosing, Multidisciplinary care

## Abstract

Im Kindes- und Jugendalter ist in Österreich, im Gegensatz zum Erwachsenenalter, der Diabetes mellitus Typ 1 (T1D) die am häufigsten auftretende Form des Diabetes mellitus (94,2 %). Nach der Diagnosestellung sollte die Betreuung der Kinder und Jugendlichen in einem pädiatrischen Zentrum mit viel Erfahrung in pädiatrischer Diabetologie erfolgen. Eine lebenslange Insulintherapie ist notwendig, wobei diese individuell an das Alter und den Alltag der Familie angepasst werden soll. In diesem Alter wird ausdrücklich die Verwendung von Diabetestechnologie (kontinuierliche Sensorglukosemessung, Insulinpumpentherapie und automatisierte Insulindosierung [AID]) empfohlen. Eine möglichst optimale metabolische Einstellung ab Therapiebeginn verbessert die Langzeitprognose der jungen Menschen mit Diabetes. Ein wesentlicher Teil in der Betreuung ist die Schulung von jungen Menschen mit Diabetes und deren Erziehungsberechtigten von einem entsprechend ausgebildeten multidisziplinären Team, bestehend aus pädiatrischen DiabetologInnen, DiabetesberaterInnen, DiätologInnen, PsychologInnen und SozialarbeiterInnen. Die APEDÖ (Arbeitsgruppe für pädiatrische Endokrinologie und Diabetologie Österreich) und die ISPAD (International Society for Pediatric and Adolescent Diabetes) empfehlen als metabolisches Ziel für alle pädiatrischen Altersgruppen einen HbA_1c_-Wert ≤ 7,0 % (IFCC ≤ 53 mmol/mol) bzw. ≤ 6,5 rel % (IFCC ≤ 48 mmol/mol) bei Verwendung von fortgeschrittener Diabetestechnologie wie AID mit einer „time in range“ (TIR) > 70 % ohne schwere Hypoglykämien. Eine altersentsprechend normale körperliche, kognitive und psychosoziale Entwicklung sowie die Vermeidung von Akutkomplikationen (schwere Hypoglykämien, diabetische Ketoazidose), das Screening auf assoziierte Erkrankungen und die Prävention von diabetesbedingten Spätkomplikationen zum Erhalt einer hohen Lebensqualität sind die Ziele der pädiatrischen Diabetestherapie.

## Abstract (en)

In contrast to adults, type 1 diabetes mellitus (T1D) is the most frequent form of diabetes in childhood and adolescence (94.2%) in Austria. After diagnosis the management of children and adolescents with T1D should take place in highly specialized pediatric units experienced in pediatric diabetology. The lifelong substitution of insulin is the cornerstone of treatment whereby modalities need to be individually adapted for patient age and the family routine. In this age group the use of diabetes technology (glucose sensors, insulin pumps and automated insulin dosage, AID) is recommended. An optimal metabolic control right from the start of treatment is associated with an improved long-term prognosis. Diabetes education is essential for youth with diabetes and their families and needs to be performed by a multidisciplinary team consisting of pediatric diabetologists, diabetes counsellors, dieticians, psychologists and social workers. The Austrian working group for pediatric endocrinology and diabetes (APEDÖ) and the International Society for Pediatric and Adolescent Diabetes (ISPAD) recommend a metabolic goal of glycated hemoglobin (HbA1c) ≤ 7.0% (International Federation of Clinical Chemistry and Laboratory Medicine, IFCC < 53 mmol/mol) or ≤ 6.5 rel % (IFCC 48 mmol/mol) for those who have access to advanced diabetes technologies, such as AID and a time in range (TIR) of > 70% for all pediatric age groups without the occurrence of severe hypoglycemia. Appropriate age-related physical, cognitive and psychosocial development, screening for associated diseases, avoidance of acute diabetes-related complications (severe hypoglycemia, diabetic ketoacidosis) and prevention of diabetes-related late complications to ensure high quality of life are the main goals of diabetes treatment in all pediatric age groups.

## Definition

Diabetes mellitus ist eine Stoffwechselerkrankung mit unterschiedlicher Ätiologie, welche durch eine persistierende Hyperglykämie, bedingt durch eine Störung der Insulinsekretion und/oder Insulinwirkung charakterisiert ist.

## Klassifikation

Die derzeit gültige Klassifikation der American Diabetes Association (ADA) und der International Society for Pediatric and Adolescent Diabetes (ISPAD) teilt die verschiedenen Diabetesformen in Typ 1–4 ein [1, 2]. Im Kindes- und Jugendalter tritt in Österreich in 94,2 % ein Diabetes mellitus Typ 1 (T1D) auf, der aufgrund des Insulinmangels rasch zu einer diabetischen Ketoazidose (DKA) führen kann. In den letzten Jahren konnte in Österreich ein signifikanter Anstieg der DKA bei Erstmanifestation beobachtet werden. Die bedeutet, dass die Diagnose oft verzögert und sehr spät gestellt wird [3, 4]. Die Erstmanifestation eines T1D kann in jedem Kindes- und Jugendalter auftreten, auch im Säuglingsalter. Der Erkrankungsgipfel liegt im Volksschulalter, es sind aber zunehmend jüngere Kinder betroffen.

Weitere im Kindes- und Jugendalter vorkommende Diabetesformen sind Diabetes mellitus Typ 2 (T2D), spezifische Diabetes-mellitus-Typen (z. B. MODY [Maturity Onset Diabetes of the Young]), CF-related Diabetes mellitus (CFRD), Diabetes mellitus nach Transplantation, bei Kortisontherapie sowie assoziiert bei verschiedenen Syndromen (z. B. Trisomie 21, Prader-Willi-Syndrom) [2].

## Epidemiologie

Der T1D ist die häufigste Stoffwechselerkrankung im Kindes- und Jugendalter. Die Inzidenz der Erkrankung < 15 Jahren nimmt weltweit kontinuierlich zu [3, 5, 6].

Die aktuellen Daten des österreichischen Diabetes-Inzidenz-Registers in der Altersgruppe 0 bis 14 Jahre stammen aus dem Zeitraum 1999 bis 2021 [3]. Von den 5888 in diesem Zeitraum erfassten Fälle hatten 94,3 % einen T1D, 1,8 % einen T2D und 3,9 % eine andere Form des Diabetes. Nachdem in den Jahren 1989 bis 2011 ein kontinuierlicher Anstieg der T1D-Inzidenz beobachtet wurde („annual percent change“ [APC] 4,56 %, *p* < 0,001) folgte in den Jahren 2012 bis 2020 eine Plateauphase (APC 0,78 %, *p* = 0,379). Im Jahr 2021 wurde mit 28,6/100.000/Jahr (95 % CI: 25,7–31,6) die bisher höchste österreichische standardisierte T1D-Inzidenzrate beobachtet. Diese Zunahme im Jahr 2021 fällt zeitlich, mit der globalen COVID-19-Pandemie zusammen. Im Inzidenz-Register sind keine Infektionen oder Impfungen erfasst, daher kann daraus kein direkter kausaler Zusammenhang gezogen werden.

Zum ersten Mal konnte ein statistisch signifikanter Anstieg der T2D-Inzidenzrate beobachtet werden (1999–2021: APC 3,47 %, *p* = 0,014). Andere Diabetesformen kommen in der Altersgruppe bis < 15 Jahren aber doppelt so oft vor wie der T2D [3].

## Klinische Symptome

Beim T1D im Kindes- und Jugendalter treten meist klassische Symptome wie Polyurie, Enuresis, Polydipsie, Gewichtsverlust, Müdigkeit, Konzentrationsstörungen, Sehstörungen, Verhaltensauffälligkeiten oder Soorinfektionen auf, wobei die Dauer dieser Symptome meist kurz (Tage bis Wochen) ist. Wird die Diagnose spät gestellt, dann treten auch Symptome einer diabetischen Ketoazidose auf (u. a. vertiefte Atmung, Übelkeit, Erbrechen, Bewusstseinstrübung). Je jünger das Kind ist, desto schwieriger kann es sein, die Symptome einer Diabetesmanifestation zuzuordnen. Trotz einer bereits hohen DKA-Prävalenz bei T1D-Diagnose in Österreich nahm diese zwischen 2012 und 2020 weiter zu und lag im Mittel bei 43,6 % [95 % CI: 41,6; 45,7]. Besonders hoch ist die DKA-Prävalenz bei Kleinkindern unter 2 Jahren, wo 72 % betroffen sind und davon ein Drittel mit schwerer DKA behandelt werden muss [4].

## Diagnosekriterien für einen Diabetes mellitus

Die Diagnose eines T1D im Kindesalter wird meist anhand der typischen Symptome, einer Harnuntersuchung, der Blutzucker- und HbA_1c_-Bestimmung gestellt, zusätzlich sind bei rund 85 % der Kinder und Jugendlichen mit T1D diabetesspezifische Autoantikörper nachweisbar: Inselzell-Antikörper (ICA), Tyrosinphosphatase-ähnliche Protein-2-Antikörper (IA-2A), Insulin-Autoantikörper (IAA), Glutamatdecarboxylase-Antikörper (GADA) und Zinktransporter-8-Antikörper (ZnT8-A). Es gelten in der Pädiatrie die gleichen Diagnosekriterien wie bei Erwachsenen, lediglich die Dosis der Glukosebelastung beim oralen Glukosetoleranztest (oGTT) ist gewichtsbedingt unterschiedlich. Ein oGTT ist bei Kindern und Jugendlichen mit T1D nur selten notwendig, spielt aber bei der Diagnose seltenerer Diabetesformen (z. B. T2D, MODY, CF-related DM) eine wichtige Rolle (Tab. 1).

**Tab. 1:** Diagnosekriterien Diabetes mellitus im Kindes- und Jugendalter (Update 2026) [1, 2]

HbA_1c_ > 6,5 % (IFCC > 48 mmol/mol) (DCCT-standardisiertes Labor)
Oder
Nüchtern-Plasma-Glukose ≥ 126 mg/dl (mindestens 8 h keine Kalorienaufnahme)
Oder
2‑h-Plasma-Glukose beim oGTT ≥ 200 mg/dl (der oGTT soll mit einer Glukosebelastung von 1,75 g/kg Körpergewicht, maximal 75 g durchgeführt werden)
Oder
Klassische diabetesspezifische Symptome oder hyperglykämische Krise mit einer Plasmaglukose ≥ 200 mg/dl

Bei klassischen Symptomen und Hyperglykämie und/oder Glukosurie/Ketonurie sollten die Kinder/Jugendlichen umgehend an eine Kinderabteilung mit ausreichend Erfahrung in der Behandlung von Kindern mit Diabetes und Expertise in pädiatrischer Diabetologie überwiesen werden. Der Anstieg der DKA-Rate bei Erstmanifestation weist auf eine verzögerte Diagnosestellung hin. Fast jedes zweite Kind mit Diabetesmanifestation wird in Österreich zu spät diagnostiziert [4].

Bei Kindern und Jugendlichen mit fehlender klinischer Symptomatik, aber nachgewiesener Hyperglykämie und/oder Glukosurie (dies kann z. B. transient im Rahmen eines Infektes auftreten) sollten eine Kontaktaufnahme sowie eine weitere Abklärung in einem Zentrum für pädiatrische Diabetologie erfolgen.

### Stadien des Diabetes mellitus Typ 1 [7]

Die ISPAD definiert Typ-1-Diabetes in der aktuellen Leitlinie von 2024 als eine progressive Autoimmunerkrankung mit folgenden Stadien [7]:

### „At risk“

Individuen, bei denen ein einzelner β‑Zell-spezifischer Autoantikörper festgestellt wird, werden in der aktuell gültigen Klassifikation als „at risk“ eingestuft.

Sobald 2 oder mehr diabetesspezifische Autoantikörper nachgewiesen werden, wird folgende Stadieneinteilung definiert:

### Stadium 1

Dieses Stadium ist gekennzeichnet durch die Präsenz von mindestens 2 diabetesspezifischen Autoantikörpern. Die Betroffenen weisen normoglykämische Werte und keine klinischen Symptome auf.

### Stadium 2

Dieses Stadium ist gekennzeichnet durch die Präsenz von mindestens 2 diabetesspezifischen Autoantikörpern. Es kommt es zur Dysregulation der Glukosehomöostase, gekennzeichnet durch eine gestörte Glukosetoleranz. Klinische Symptome fehlen weiterhin, jedoch ist die β‑Zell-Funktion deutlich beeinträchtigt.

### Stadium 3

Das klinische Manifestationsstadium, in dem die Insulinsekretion so weit reduziert ist, dass Symptome (Polyurie, Polydipsie, Gewichtsverlust, Ketoazidose) auftreten. Die Diagnose eines manifesten T1D erfolgt.

Diese Stadieneinteilung ermöglicht eine systematische Klassifikation des Krankheitsverlaufs, unterstützt frühzeitige Diagnostikstrategien und kann potenziell präventive Interventionen im präklinischen Stadium fördern [7].

## Therapie und Ziele

Die Betreuung und Behandlung von Kindern und Jugendlichen mit Diabetes mellitus sollte grundsätzlich in einem Zentrum für pädiatrische Diabetologie bzw. einer Kinderabteilung mit ausreichender Erfahrung in pädiatrischer Diabetologie erfolgen. Diese Zentren benötigen eine ausreichende personelle Besetzung mit einem multidisziplinären Team [8].

### Ziele

Eine altersentsprechend normale körperliche, kognitive und psychosoziale Entwicklung sowie die Vermeidung von Akutkomplikationen (schwere Hypoglykämien, diabetische Ketoazidosen) und die Prävention von diabetesbedingten Spätkomplikationen (diabetische Retinopathie, diabetische Nephropathie, diabetische Neuropathie u. a.) zum Erhalt einer hohen Lebensqualität sind die Ziele der pädiatrischen Diabetestherapie.

Dies beinhaltet eine regelmäßige Kontrolle des Längen- und Gewichtsverlaufs, des Pubertätsstatus sowie die Durchführung regelmäßiger Screeninguntersuchungen auf das Vorliegen von anderen chronischen Erkrankungen im Frühstadium und diabetesassoziierter Erkrankungen (z. B. Zöliakie, Schilddrüsenerkrankungen u. a.).

Die österreichische Arbeitsgruppe für pädiatrische Endokrinologie und Diabetologie (APEDÖ) wie auch die ISPAD empfehlen als Ziel für die metabolische Einstellung bei Kindern und Jugendlichen, die mit AID behandelt werden, HbA_1c_-Werte ≤ 6,5 % (≤ 48 mmol/mol) [9]. Es gilt, den niedrigsten HbA_1c_-Wert anzustreben, welcher ohne schwere Hypoglykämien zu erreichen ist.

**Abb. 1:**
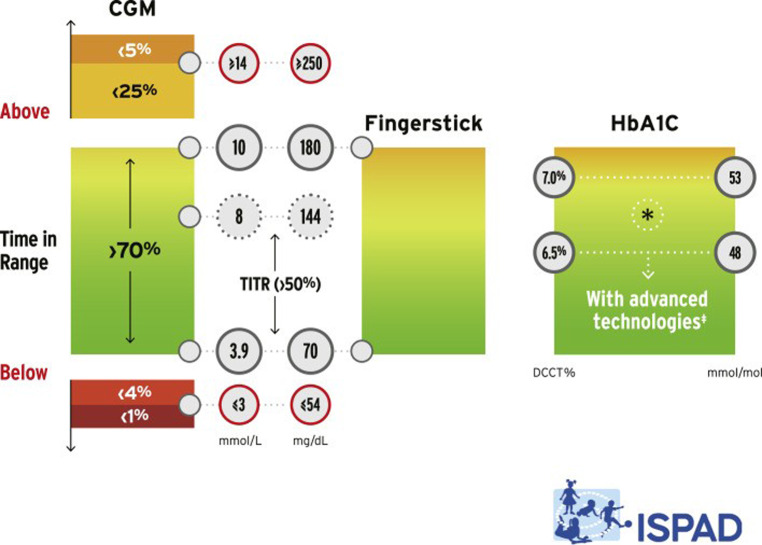
Glykämische Ziele der ISPAD 2024 [9]

Da der Großteil der Kinder und Jugendlichen mit Diabetes mittels Glukosesensoren versorgt ist, können die ambulanten Glukoseprofile (Auswertungszeitraum mindestens 14 Tage) zur Beurteilung der metabolischen Einstellung herangezogen werden. Die Glukoseprofile geben im Gegensatz zum HbA_1c_ mehr Information zur Verteilung von Hypo- und Hyperglykämien. Der Begriff „time in range“ (TIR = Zeit im Zielbereich), d. h. der Prozentsatz an Zeit mit Sensorglukosewerten im Bereich zwischen 70–180 mg/dl, hat zunehmend an Bedeutung gewonnen und ist zu einer neuen Messgröße zusätzlich zum HbA_1c_ geworden. Mehr als 70 % eines Tages sollten in diesem Bereich verbracht werden. Weitere Ziele sind, weniger als 4 % des Tages unter 70 mg/dl (3,9 mmol/l), weniger als 1 % unter 54 mg/dl (3 mmol/l) und < 25 % > 180 mg/dl (10 mmol/l) bzw. < 5 % > 250 mg/dl (13,9 mmol/l) zu verbringen [10]. Die Zeit im Zielbereich korreliert mit dem HbA_1c_-Wert hinsichtlich des Risikos für Folgeerkrankungen [11] Wird eine noch engere glykämische Kontrolle angestrebt, kann das Maß „time in tight range“ (TITR) mit Glukosewerten zwischen 70–140 mg/dl orientierend verwendet werden, gelegentlich auch synonym als „time in near normoglycemia“ (TING) bezeichnet. Die Festlegung eines Ziels, mindestens 50 % des Tages zwischen 70–140 mg/dl („time in tight range“ [TITR]) zu verbringen, ist derzeit in Diskussion. Bei blutiger Glukosemessung sollten ebenfalls 70 % der Werte zwischen 70 und 180 mg/dl liegen, das Ziel für die Nüchtern-Glukose liegt zwischen 70 und 144 mg/dl ([9]; Abb. 1).

Zusammenfassung der metabolischen Ziele laut ISPAD-Leitlinie: Die niedrigeren HbA_1c_-Werte gelten für jene Menschen mit Diabetes, die technische Diabetestherapie, insbesondere AID-Systeme verwenden.

## Kontinuierliche Behandlung bei Typ-1-Diabetes mellitus Stadium 3

### Insulintherapie

Die Insulintherapie bei T1D beginnt bei manifester, symptomatischer Dysglykämie und Hyperglykämie (Stadium 3). Der Insulinbedarf kann in unterschiedlichen Phasen der Erkrankung variieren, wobei während der Pubertät besonders hohe Insulindosen benötigt werden, während es in der Remissionsphase oft einen sehr geringen Insulinbedarf gibt. Es wird empfohlen, bei Kindern und Jugendlichen eine individualisierte, intensivierte Insulintherapie einzuleiten, wobei der technischen Diabetestherapie mit AID-Systemen der Vorzug zu geben ist [9]. Grundsätzlich stehen für die Insulintherapie folgende Möglichkeiten zur Verfügung: Basis-Bolustherapie, Insulinpumpentherapie, sensorunterstützte Pumpentherapie sowie Automated-Insulin-Delivery(AID)-Systeme, häufig auch als Closed-Loop-Systeme bezeichnet. Zur prandialen Insulinsubstitution und zur Korrektur sollten kurz wirksame Insuline, schnell oder ultraschnell wirksame Insulinanaloga, welche sich hinsichtlich ihres Wirkbeginns und der Wirkdauer unterscheiden, verwendet werden. In Insulinpumpen sollten vorzugsweise schnell oder ultraschnell wirksame Insulinanaloga Verwendung finden [12]. Bei Kleinkindern ist gelegentlich aufgrund des geringen Insulinbedarfs die Verwendung von verdünntem Insulin (U10/U20/U50) notwendig. Die Verdünnung kann mittels physiologischer Kochsalzlösung oder mit von der pharmazeutischen Industrie zur Verfügung gestelltem Verdünnungsmedium hergestellt werden. Die Haltbarkeit der verdünnten Insuline ist begrenzt (4 Wochen). Die Verdünnung selbst sollte vorzugsweise von Apotheken hergestellt werden. Als Basalinsuline werden überwiegend lang oder ultralang wirksame Insulinanaloga (seltener NPH-Insuline) zur Substitution des basalen Insulinbedarfs verwendet. Lang wirksame Insuline finden bei der Pumpentherapie keine Verwendung, da der Basalbedarf durch die kontinuierliche Abgabe von schnell wirksamen Insulinanaloga (Basalrate) abgedeckt wird. Die Insulinpumpentherapie sollte allen Kindern, Jugendlichen und jungen Erwachsenen mit T1D unabhängig von Alter, Diabetesdauer und glykämischer Einstellung empfohlen werden, wobei die modernste Technologie, die verfügbar und für die jeweilige Altersgruppe zugelassen ist, angeboten werden sollte. Die Wahl der Geräte und Systeme sollte unter Berücksichtigung individueller Bedürfnisse und Vorlieben erfolgen [13].

Der Therapie mit AID-Systemen (automatische Insulindosierung), bestehend aus den 3 Komponenten Glukosesensor, Insulinpumpe und Algorithmus, ist aufgrund der besseren metabolischen Einstellung der Vorzug zu geben. Der Einsatz der AID-Systeme sollte bereits bei Manifestation der Insulinbehandlungspflichtigkeit erfolgen. Die Anwendung von AID-Systemen gilt in der Pädiatrie als dringend empfohlen, da die Evidenz für eine bessere Zeit im Zielbereich, einer Reduktion von Hyperglykämien, einer Verbesserung der metabolischen Einstellung und der Verbesserung der Lebensqualität eindeutig gegeben ist. Diese Benefits liegen vor, ohne dabei das Risiko für Hypoglykämien zu erhöhen [9].

Alle derzeit in Österreich erhältlichen und im Kindesalter zugelassenen Systeme (ab 1. Lebensjahr YpsoPump [mylife YpsoPump, Bern, Schweiz] und CamAPS [CamDiab, Cambridge, UK], ab 2. Lebensjahr Medtronic 780 G [Medtronic, Northridge, CA 91325, USA]) sind AID-Systeme, das bedeutet, dass für die Mahlzeiten manuelle Boli abgegeben werden müssen [13].

Eine gute metabolische Einstellung von Beginn an ist prognostisch essenziell [14–16], daher ist es notwendig, eine individualisierte, altersadäquate Therapie anzuwenden, um eine hohe Therapiezufriedenheit und ein gutes metabolisches Outcome zu erreichen.

### Glukosemessung

Eine regelmäßige Glukosemessung entweder durch Blutzuckerbestimmung in kapillarem Blut aus der Fingerbeere oder mittels kontinuierlicher subkutaner Glukosemessung (CGM) muss begleitend zur Insulintherapie bei allen Kindern durchgeführt werden. Die blutige Messung der Glukosewerte wurde in den vergangenen Jahren weitestgehend durch die CGM verdrängt [17]. Bei alleiniger Blutzuckermessung mittels Kapillarblut ist eine Häufigkeit von 6 bis 10 Messungen täglich empfohlen. Die Messung sollte jeweils *vor* den Mahlzeiten und in der Nacht stattfinden. Bei Anwendung von Glukosesensoren können je nach System blutige Messungen zur Kalibration oder zur Kontrolle bei nichtplausiblen Werten empfohlen sein.

CGM-Systeme sind ab dem 1. Lebensjahr anwendbar, wobei sich die altersabhängige Zulassung von Gerät zu Gerät unterscheidet. Jedenfalls ist die Anwendung von CGM schon ab Beginn der Insulintherapie bei Kindern, Jugendlichen und jungen Erwachsenen mit T1D indiziert. Auch junge Menschen mit T2D profitieren von CGM-Systemen. Die Liegedauer der Sensoren unterscheidet sich je nach Gerät.

Eine regelmäßige Dokumentation der Glukosewerte, Insulindosen und Kohlenhydrateinheiten erscheint im Alltag hilfreich und kann weitestgehend mit elektronischen Hilfsmitteln erfolgen. Alle diabetesrelevanten Geräte (Pumpe, Glukosemessgeräte, Sensoren) sollten im Rahmen der ärztlichen Kontrolle ausgelesen, standardisiert ausgewertet und analysiert werden, um therapeutische Maßnahmen ableiten und empfehlen zu können. Die gemeinsame Datenanalyse von Anwender und Diabetesteam ist essenziell. Diese beinhaltet auch fortlaufende Schulungen der Familien und des Fachpersonals in der Anwendung von CGM und in der Dateninterpretation, um Ergebnisse erfolgreich zu optimieren [17].

Weitere Details und Empfehlungen s. ÖDG Leitlinie Technologie.

### Therapie bei Diabetes mellitus Typ 2 (T2D)

Zur Therapie des T2D bei Kindern und Jugendlichen wird eine Lebensstilmodifikation in Kombination mit einer medikamentösen Therapie empfohlen [18]. Für eine medikamentöse Therapie des T2D sind derzeit Metformin (ab dem 10. Lebensjahr), Insulin, GLP-1-Analoga (Liraglutid, Dulaglutid) und SGLT-2-Inhibitoren (Empagliflozin, Dapagliflozin) ab dem 10. Lebensjahr zugelassen [18].

Als Ziel für die metabolische Einstellung wird auch beim T2D ein HbA_1c_-Wert ≤ 6,5 % (IFCC ≤ 48 mmol/mol) angestrebt. Ein höheres HbA_1c_-Ziel kann kurzfristig mit < 7 % (53 mmol/mol) bei Jugendlichen zu Beginn der Therapie bzw. in Einzelfällen mit starker Beeinträchtigung der Lebensqualität (z. B. bei signifikanter Hypoglykämie) akzeptiert werden [18]. Die Nüchternglukoseziele liegen bei 70–110 mg/dl (4–6 mmol/l). Die postprandialen Blutzuckerziele liegen bei 70–140 mg/dl (4–8 mmol/l). Bei Verwendung von CGM sollten die etablierten CGM-Ziele für T1D übernommen werden, da für T2D bisher keine Ziele festgelegt wurden [18].

Die initiale Behandlung sollte von klinischer Symptomatik, Schweregrad der Hyperglykämie bzw. Bestehen einer Ketose/Ketoazidose abhängig gemacht werden [18].

1. Bei metabolisch stabilen, asymptomatischen Patienten mit einem initialen HbA_1c_-Wert < 8,5 % (69,4 mmol/mol) soll mit einer Monotherapie mit Metformin begonnen werden.
2. Bei Patienten mit einem initialenHbA_1c_-Wert ≥ 8,5 % (69 mmol/mol) ohne Vorliegen einer Ketose/Azidose umfasst die Anfangsbehandlung Basalinsulin (Startdosis 0,25–0,5 Einheiten/kg). Parallel dazu sollte mit einer Metformin-Therapie bis zur maximal verträglichen Menge von bis zu 2000 mg einschleichend gestartet werden. Zu beachten ist allerdings, dass eine Insulintherapie mit dem Risiko einer unerwünschten Gewichtszunahme und, wenn auch selten, mit Hypoglykämien einhergehen kann.
3. Bei Vorliegen einer Ketoazidose und/oder eines hyperosmolaren hyperglykämischen Syndroms (HHS) sollte intravenöses Insulin begonnen werden.

Bei Ausschleichen der Insulintherapie sollte, auch wenn keine diabetesspezifischen Autoantikörper nachgewiesen werden konnten, an die Möglichkeit des Vorliegens eines T1D gedacht werden, insbesondere wenn bei der Diabetesdiagnosestellung eine DKA vorlag.

- Erhaltungstherapie (2 bis 3 Monate nach Diagnose)
  - Wenn ein HbA_1c_ von < 6,5 % (53 mmol/mol) nicht erreicht wird, sollten GLP-1-RA- und/oder SGLT-2-Inhibitoren in Betracht gezogen werden [A] und individuell an die Bedürfnisse und Präferenzen der Jugendlichen mit Typ-2-Diabetes angepasst werden [E].
- Deutliche und anhaltende HbA_1c_-Anstiege trotz Kombinationstherapie erfordern eine Insulintherapie. Das Basalinsulin sollte maximiert werden, und bei weiterhin nicht erreichten Blutzuckerzielen sollte ein kurz wirksames prandiales Insulin hinzugefügt werden.

Bei der Klassifikation zwischen Typ-1- und Typ-2-Diabetes sollten mehrere Faktoren berücksichtigt werden: das Vorliegen einer Adipositas (BMI > 85. Perzentile), der Zeitpunkt der Manifestation (prä- oder postpubertär), das Vorhandensein metabolischer Komorbiditäten sowie die aktuelle klinische Symptomatik (z. B. Ketoazidose). Im Falle fehlender diabetesspezifischer Autoantikörper und fehlender klinischer Hinweise auf Insulinresistenz sowie klinische und anamnestische Hinweise auf urogenitale Auffälligkeiten, neonatalen Diabetes, positiver Familienanamnese und/oder stabiler isolierter Nüchtern-Hyperglykämie sollte eine genetische Untersuchung auf MODY eingeleitet werden.

### Ernährung

Die Ernährungsschulung und das Einhalten einer kohlenhydratberechnenden Kost sind eine wichtige Grundvoraussetzung für eine gute metabolische Einstellung. Die Schulung über die Berechnung der Nahrung (insbesondere der Kohlenhydrate) und deren Wirkung auf den Blutzucker sollte von DiätologInnen durchgeführt werden. Es sollte dabei auf kulturelle Ernährungsgewohnheiten Rücksicht genommen werden.

Neben einer altersgerechten Kalorien- und Energiezufuhr muss ein ausreichender Anteil an Kohlenhydraten (50–55 %) zur Sicherstellung eines regulären altersentsprechenden Wachstums zugeführt werden [21].

Der prandiale Insulinbedarf wird durch die Berechnung der Kohlenhydrate ermittelt. Kohlenhydrate wurden im deutschsprachigen Raum historisch bedingt lange als Broteinheit (BE = 12 g Kohlenhydrate) oder Kohlenhydrateinheit (KE = 10 g Kohlenhydraten) angegeben, wobei sich in den letzten Jahren die Angabe in „Gramm Kohlenhydraten“ nicht mehr nur bei Insulinpumpentherapie im klinischen Alltag immer mehr durchsetzt.

Neben der Berechnung der Kohlenhydrate zeigt die Berücksichtigung des glykämischen Index (GI) mit Bevorzugung von Kohlenhydraten mit niedrigen GI positive Effekte auf die glykämische Kontrolle [19].

### Schulung

Eine altersangepasste, strukturierte Diabetesschulung ist integrativer Bestandteil der therapeutischen Bemühungen und Voraussetzung für ein funktionierendes Diabetesmanagement zu Hause. Die Diabetesschulung umfasst alle in dieser Behandlungsempfehlung aufgelisteten Aspekte der pädiatrischen Diabetestherapie. Eltern bzw. Betreuungspersonen aus dem sozialen Umfeld müssen ins Diabetesmanagement eingebunden werden.

Das multidisziplinäre Schulungsteam sollte pädiatrische DiabetologInnen, DiabetesberaterInnen, DiätologInnen, PsychologInnen sowie SozialarbeiterInnen umfassen.

### Psychologische Betreuung

Dem multidisziplinären Behandlungsteam sollten PsychologInnen mit Expertise in der Betreuung von Kindern und Jugendlichen mit Diabetes angehören, welche bei der Erfassung der psychosozialen Situation der PatientInnen/Familien im Rahmen dieser chronischen Erkrankung eine wichtige Rolle einnehmen und bei Bedarf auch spezifische Interventionen durchführen können. Der Erstkontakt mit Kind und Familie erfolgt idealerweise bei der Diabeteserstmanifestation, die weitere Betreuung im Verlauf umfasst psychologische Beratung, Früherkennung von psychosozialen Problemen, psychologische Behandlung und Unterstützung bei der Etablierung einer erweiterten Betreuung bei psychosozialen Problemen.

Ein höheres Risiko für psychische Komorbiditäten ist bei T1D bekannt (z. B. Essstörungen, Insulinmanipulation, Depression, Angststörung, ADHS u. a.), und diese können Einfluss auf die Diabetestherapie und damit auch auf die metabolische Kontrolle und das Auftreten von Komplikationen nehmen [20]. Ein jährliches Screening auf Symptome psychischer Begleiterkrankungen ist daher ab dem 12. Lebensjahr jährlich empfohlen.

## Akute Komplikationen

Zu den Akutkomplikationen des T1D zählen zum einen die schwere Hypoglykämie und zum anderen die diabetische Ketoazidose (DKA).

Die schwere Hypoglykämie ist definiert als ein Ereignis mit Bewusstseinsbeeinträchtigung (Koma oder Krampfanfall), das einer Fremdhilfe bedarf.

Die Behandlung der schweren Hypoglykämie erfolgt mit Glukagon-Nasenspray (3 mg nasal ab dem 4. Lebensjahr) oder Glukagon-Fertigspritze (0,5–1 mg) i.m. oder s.c. (keine Altersbeschränkung). Im Kliniksetting kann eine Hypoglykämie auch mit intravenöser Glukosezufuhr von 2–3 ml/kgKG 10 % Glukose erfolgen. In jedem Haushalt mit einer Person mit T1D sollte die Notfallmedikation Glukagon (Nasenspray oder Glukagon-Fertigspritze) vorhanden sein. Die Behandlung mit Glukagon setzt eine entsprechende Schulung voraus [21].

Kleinkinder sind aufgrund der mangelnden Fähigkeit, Hypoglykämiesymptome zu äußern, und durch eine verminderte Hypoglykämiewahrnehmung einem höheren Risiko für das Auftreten einer schweren Hypoglykämie ausgesetzt.

Internationale Registerstudien konnten zeigen, dass die Inzidenz der schweren Hypoglykämien in den letzten 2 Dekaden abgenommen hat bei gleichzeitig sinkenden durchschnittlichen HbA_1c_-Werten. Eine Korrelation von niedrigen HbA_1c_-Werten und damit verbunden höherer Hypoglykämierate konnte daher nicht bestätigt werden [22–24].

Eine weitere akute Komplikation ist die diabetische Ketoazidose (DKA). In Österreich wurde im Zeitraum 2012 bis 2020 ein deutlicher Anstieg der DKA bei Diabetesmanifestation beobachtet [7]. Auch im Verlauf der Diabeteserkrankung ist eine DKA möglich, die Ursache ist immer ein Insulinmangel [25]. Die klinischen Zeichen einer DKA sind: Dehydratation, Tachykardie, Tachypnoe, Kussmaul-Atmung, Azetongeruch, Übelkeit und Erbrechen, Schläfrigkeit bis hin zum Koma. Risikofaktoren für eine DKA bei Erstmanifestation sind: jüngeres Alter, verzögerte Diagnosestellung, niedriger sozioökonomischer Status und Länder mit niedriger Diabetesinzidenz. Risikofaktoren für eine DKA bei bekanntem T1D sind v. a. absichtliches Weglassen der Insulininjektionen, Fehler beim Management von Krankheit und Fehler beim Management der Insulinpumpentherapien sowie das Vorliegen von psychischer Begleiterkrankungen (inklusive Essstörungen).

Die Therapie und Überwachung von Kindern mit DKA sollte von ÄrztInnen durchgeführt werden, die über Erfahrung in diesem Gebiet verfügen, und es muss die Möglichkeit zu einer intensivmedizinischen ärztlichen, pflegerischen und biochemischen Überwachung gesichert sein.

Die Therapieziele der DKA sind: Ausgleich der Dehydratation, Ausgleich der Azidose, Blutzuckerstabilisierung und -normalisierung und Vermeidung von Komplikationen (Hirnödem und Hypokaliämie). Die Therapie besteht aus Flüssigkeits‑, Elektrolyt- (Kalium, Phosphat) und Insulinsubstitution (über 24–48 h). Mit einer i.v.-Flüssigkeitssubstitution sollte unverzüglich, noch vor Beginn der Insulin- und Elektrolytsubstitution, in Form von 0,9 % NaCl-Lösung begonnen werden. Vor Beginn der Kaliumsubstitution sollte die Diurese bzw. Nierenfunktion gesichert werden [25].

Die Vermeidung von diesen akuten Komplikationen zählt zu den vorrangigen Zielen in der Betreuung von Kindern und Jugendlichen mit T1D.

## Langzeitkomplikationen und empfohlene Screeninguntersuchungen

Bei den Follow-up-Untersuchungen sollen routinemäßig die Körperlänge, das Körpergewicht, der Blutdruck (unter Verwendung von alters- und geschlechtsspezifischen Perzentilen), das Pubertätsstadium nach Tanner sowie die Injektionsstellen bzw. Katheterinjektionsstellen (zur Erkennung von Lipohypertrophien/Lipoatrophien und Hautirritationen) und die CGM-Stellen (zur Erkennung von Ekzemen/Hautirritationen/Abszessen) kontrolliert werden [26, 27].

Einmal im Jahr ist auch die Kontrolle der Nieren- und Leberfunktionsparameter, des Blutbildes sowie des Lipidstatus indiziert. Der HbA_1c_ sollte alle 3 Monate bestimmt werden.

Um das Risiko für mikrovaskuläre und makrovaskuläre Komplikationen zu senken, sollte eine möglichst normoglykämische Stoffwechseleinstellung angestrebt werden, um das Auftreten von diabetischen Komplikationen wie eine diabetische Retinopathie, eine Mikroalbuminurie/diabetische Nephropathie, eine Hypertonie oder eine diabetische Neuropathie bei Jugendlichen zu verhindern [28].

Ein jährliches Screening auf mikrovaskuläre Komplikationen wie Nephropathie (Morgenharn: Albumin/Kreatinin-Ratio), Retinopathie (Fundoskopie) und Neuropathie (klinische Untersuchung auf Sensibilität, Vibrationsempfinden und Reflexe) wird jährlich ab der Pubertät bzw. dem 11. Lebensjahr und 2 bis 5 Jahren Diabetesdauer empfohlen [28].

Ein Screening auf makrovaskuläre Komplikationen und Risikofaktoren (Lipidstatus) sollte kurz nach Diagnosestellung (nach Stabilisierung der Stoffwechsellage) bei allen Kindern, die älter als 11 Jahre sind, durchgeführt werden. Sind die Lipidwerte normal, genügen 3‑jährige Screeninguntersuchungen nach dem 11. Lebensjahr. Bei einer Familienanamnese auf Hypercholesterinämie oder frühen kardiovaskulären Erkrankungen sollte das Screening ab dem 2. Lebensjahr beginnen. Der Blutdruck sollte mindestens einmal jährlich, idealerweise bei jeder Visite kontrolliert werden [28].

Bei Verdacht auf eine arterielle Hypertonie sollte eine 24-h-Blutdruckmessung durchgeführt werden (unter Verwendung von alters- und geschlechtsspezifischen Normwerten) [30]. Zusätzlich zu einer Lifestyleintervention (Reduktion von Salzkonsum, tierische Fette sowie zuckerhaltige Getränke und Süßigkeiten, Steigerung der körperlichen Aktivität und Reduktion von sitzenden Tätigkeiten) werden ACE-Hemmer zur Senkung eines erhöhten Blutdrucks im Kindes- und Jugendalter empfohlen und haben sich als sichere und effektive Therapie in Kurzeitstudien erwiesen. Der klinische Benefit von Angiotensin-II-Rezeptorantagonisten ist ähnlich wie bei ACE-Hemmern, wobei ihre Verwendung in der Kinder- und Jugendheilkunde nicht so weit verbreitet ist. Aufgrund des teratogenen Potenzials sollte vor Beginn einer ACE-Hemmer-Therapie bei Mädchen eine ausführliche Aufklärung erfolgen und bei Bedarf eine effektive Verhütungsmethode implementiert werden. Des Weiteren vermindern ACE-Hemmer die Progression von einer Mikroalbuminurie zur Makroalbuminurie und erhöhen die Regressionsrate zur Normoalbuminurie [28, 29].

Bei Vorliegen einer Hypercholesterinämie sollte initial eine Lifestyleintervention mit Bewegungssteigerung und Implementierung einer fettmodifizierten Ernährung erfolgen. Sollte trotz 6‑monatiger Lifestyleintervention das LDL-Cholesterin > 130 mg/dl sein, ist ab dem 10. Lebensjahr der Beginn einer Statintherapie empfohlen. Kurzzeitstudien haben gezeigt, dass Simvastatin und Pravastatin bei Kindern effektiv und sicher in der Therapie der Hypercholesterinämie sind [30]. Es wurden keine signifikanten Nebenwirkungen im Hinblick auf Wachstum, Pubertätsfortschritt, endokrine Funktion sowie Leber und Muskelenzyme beobachtet, jedoch sollte ein Augenmerk auf Symptome gelegt werden, die die Leber sowie die Muskeln oder das Bindegewebe betreffen, da das Risiko einer Rhabdomyolyse bei Anwendung von Statinen erhöht ist. Bei Statinen muss aufgrund der potenziell teratogenen Wirkung bei Mädchen eine ausführliche Aufklärung erfolgen und ggf. eine effektive Verhütungsmethode implementiert werden [28].

Bei an T2D erkrankten Patienten sollte bereits zum Diagnosezeitpunkt ein vollständiges Screening auf mikro- und makrovaskuläre Komplikationen erfolgen [30].

## Assoziierte Erkrankungen

Kinder und Jugendliche mit T1D haben ein höheres Risiko, weitere Autoimmunerkrankungen zu entwickeln. Die häufigsten assoziierten Autoimmunerkrankungen sind die Autoimmunthyreoiditis (AIT) und die Zöliakie (CD) [26].

Bis zu 29 % der Menschen mit T1D haben bei T1D-Erstmanifestation positive Schilddrüsenantikörper, welche prädiktiv für die Entwicklung einer AIT sind, 3–8 % davon entwickeln eine AIT wobei die meisten eine Hypothyreose haben. Die AIT ist die häufigste assoziierte Autoimmunerkrankung und kommt bei Mädchen häufiger vor. Oft manifestiert sie sich in der Pubertät und ist mit einer längeren Diabetesdauer assoziiert [26, 27].

Ein Screening auf assoziierte AIT (mittels TSH, fT4, fT3 und TPO- und Tg-AK) wird bei Erstmanifestation (nach klinischer Stabilisierung) und danach alle 2 Jahre bei asymptomatischen Patienten empfohlen. Bei positiven Schilddrüsen-AK bei Erstmanifestation oder einer positiven Familienanamnese auf AIT sollte jährlich gescreent werden. Auch bei Vorliegen einer Struma, geringerer Wachstumsgeschwindigkeit oder klinischen Symptomen für eine Schilddrüsenfunktionsstörung sollte früher gescreent werden [26].

Die Hyperthyreose kommt bei Kindern und Jugendlichen mit T1D deutlich seltener vor als die Hypothyreose, jedoch häufiger als in der Normalbevölkerung. Die Prävalenz wird mit 0,5–6 % angegeben.

Die Prävalenz der Zöliakie (CD) wird bei Kindern und Jugendlichen mit T1D mit 1–16,4 % angegeben. Das CD-Risiko ist invers mit dem Alter bei Diabeteserstmanifestation assoziiert, wobei das größte Risiko bei denjenigen besteht, bei denen der Diabetes vor dem fünften Lebensjahr diagnostiziert wurde. Die klassischen Symptome der Zöliakie, wie z. B. Gedeihstörung, oder gastrointestinale Symptome sind eher selten, meist sind die Patienten asymptomatisch [26].

Ein Screening auf Zöliakie sollte mittels Transglutaminase-IgA-Antikörper (tTG IgA) und/oder Endomysium-Antikörper (EMA) und zusätzlichem Ausschluss eines IgA-Mangels durchgeführt werden. Bei nachgewiesenem IgA-Mangel ist das Zöliakiescreening mittels IgG-basierten Tests (tTG-IgG und/oder EMA-IgG) empfohlen. Das Screening auf Zöliakie wird im ersten Jahr der Diagnosestellung und danach in 2‑ bis 5‑jährigen Intervallen empfohlen. Bei klinischen Symptomen oder erstgradigen Verwandten mit Zöliakie sollte öfter gescreent werden. Genetische Parameter wie HLA-DQ2 und HLA-DQ8 sind bei Patienten mit T1D häufig positiv und werden deshalb nicht als Screeninguntersuchung empfohlen [26].

Kinder mit positiven CD-Antikörpern sollten an einer pädiatrischen Gastroenterologie vorgestellt werden. Die aktuellen ESPGHAN Guidelines aus 2020 empfehlen, dass bei PatientInnen mit hochpositiven tTG-AK (> 10-Fache des oberen Normbereichs) auf eine Dünndarmbiopsie verzichtet werden kann [30]. In diesen neuen Guidelines wird aber nicht speziell auf die Gruppe der Kinder und Jugendlichen mit T1D eingegangen.

Bei Kindern mit Symptomen und hochpositiven tTG-AK (> 10-Fache des oberen Normbereichs) und positiven EMA kann in Absprache mit der pädiatrischen Gastroenterologie und der Familie eventuell auf eine Biopsie verzichtet werden [28, 29]. Nach Einführung der glutenfreien Diät (GFD) müssen die Symptome verschwinden.

Bei asymptomatischen Kindern ist die Evidenz für einen biopsiesparenden Ansatz bei Kindern mit T1D begrenzt und wurde in den jüngsten europäischen Leitlinien nicht berücksichtigt. Die Auswirkungen einer lebenslangen glutenfreien Diät bei einer Person, die sowohl Diabetes als CD ohne Symptome hat, ist eine wichtige Überlegung, und die Entscheidung, eine Duodenalbiopsie zur Bestätigung einer gastrointestinalen Pathologie durchzuführen, sollte mit den Eltern und dem Kind besprochen werden [27].

Die APEDÖ empfiehlt derzeit bei asymptomatischen Kindern mit T1D und positiven tTG-AK (unabhängig von der Höhe der AK) weiterhin die Duodenalbiopsie, um anhand der Marsh-Klassifikation die Diagnose zu bestätigen [27] Nach Bestätigung der CD-Diagnose sollten Kinder und Jugendliche mit T1D und CD von einem/r erfahrenen pädiatrischen DiätologIn bzgl. GFD geschult werden, und sowohl Betroffene als auch ihr Diabetesteam sollten wachsam sein, da sich der Insulinbedarf während des Übergangs zur GFD ändern kann.

Weitere Autoimmunerkrankungen wie Autoimmungastritis, Morbus Addison, rheumatoide Arthritis, chronisch entzündliche Darmerkrankungen, Vitiligo oder Polyendokrinopathien sind seltener. Bei entsprechenden klinischen Symptomen sollten DiabetologInnen aber auch an die Möglichkeit dieser selteneren Autoimmunerkrankungen denken [26].

## Transition

Im Alter von 18 bis 19 Jahren bzw. mit Abschluss der Schulausbildung/Lehre sollten die jungen Erwachsenen mit Diabetes zur langfristigen Betreuung an FachärztInnen für Innere Medizin an Abteilungen für Erwachsenenmedizin übergeben werden. Diese Transition soll flexibel, je nach „Reife“ des Jugendlichen, geordnet und im Idealfall im Rahmen einer Transitionsklinik erfolgen. Die Transition sollte möglichst frühzeitig besprochen und individuell geplant werden, und es darf keine „Lücke“ in der Betreuung entstehen. Mit der Vorbereitung (einschließlich Beratung über Diabetes, Selbstmanagement, Diabeteskontrollen, Komplikationen, Unterschiede zwischen pädiatrischen und erwachsenen Systemen und Beurteilung der Bereitschaft durch den/die Pädiater:in) sollte idealerweise in den frühen Jugendjahren, mindestens aber 1 Jahr vor Transfer begonnen werden. Besonderer Fokus soll auf Selfmanagement und Selbstverantwortung gelegt werden. Eine schriftliche Zusammenfassung (Medikation, Akut- und Spätkomplikationen, assoziierte Erkrankungen, Stoffwechselkontrolle …) soll vorliegen. Spezielle Transitionskliniken und internistische Kliniken, die auf die Bedürfnisse der Jugendlichen eingehen und bei denen eine Verbindung zwischen pädiatrischem und internistischem Zentrum besteht, haben sich bewährt [31]. Rückmeldungen nach erfolgreicher Transition an den/die Pädiater:in sind wünschenswert.

## Interessenkonflikt

B. Rami-Merhar hat in den letzten 36 Monaten von folgenden Firmen Honorare/Forschungsgelder erhalten: Eli Lilly, Medtronic, Insulet, Abbott, Sanofi., E. Fröhlich-Reiterer hat in den letzten 36 Monaten von folgenden Firmen Vortragshonorare, Forschungsgelder und Honorare für Beratungstätigkeit erhalten: Medtronic, Novo Nordisk, Eli Lilly, Sanofi und Merck., S.E. Hofer erklärt in den letzten 3 Jahren Vortragshonorare der Firmen E. Lilly, Sanofi und Ypsomed erhalten zu haben., K. Nagl hat in den letzten 26 Monaten von folgenden Firmen Honorare erhalten Eli Lilly, Sanofi., M. Fritsch erklärt, in den letzten 3 Jahren Vortragshonorare der Firmen E. Lilly, Sanofi und Rhythm Pharmaceuticals erhalten zu haben., D. Meraner gibt an, dass kein Interessenkonflikt besteht.
